# Genotoxic Potential and Physicochemical Parameters of Sinos River, Southern Brazil

**DOI:** 10.1155/2013/209737

**Published:** 2013-10-29

**Authors:** Madalena C. S. Scalon, Ciliana Rechenmacher, Anna Maria Siebel, Michele L. Kayser, Manoela T. Rodrigues, Sharbel W. Maluf, Marco Antonio S. Rodrigues, Luciano Basso da Silva

**Affiliations:** ^1^Programa de Pós-graduação em Qualidade Ambiental, Universidade Feevale, 93352-000 Novo Hamburgo, RS, Brazil; ^2^Curso de Ciências Biológicas, Universidade Feevale, 93352-000 Novo Hamburgo, RS, Brazil; ^3^Curso de Biomedicina, Universidade Feevale, 93352-000 Novo Hamburgo, RS, Brazil; ^4^Laboratório de Citogenética, Serviço de Genética Médica, Hospital de Clínicas de Porto Alegre, 90035-903 Porto Alegre, RS, Brazil; ^5^Programa de Pós-graduação em Qualidade Ambiental, Grupo de Pesquisa em Tecnologias Ambientais, 93352-000 Novo Hamburgo, RS, Brazil; ^6^Programa de Pós-graduação em Qualidade Ambiental, Grupo de Pesquisa em Indicadores de Qualidade Ambiental, Universidade Feevale, RS 239, No. 2755, 93352-000 Novo Hamburgo, RS, Brazil

## Abstract

The present study aimed to evaluate the physicochemical parameters and the genotoxic potential of water samples collected in the upper, middle, and lower courses of the Sinos River, southern Brazil. The comet assay was performed in the peripheral blood of fish *Hyphessobrycon luetkenii* exposed under laboratory conditions to water samples collected in summer and winter in three sampling sites of Sinos River. Water quality analysis demonstrated values above those described in Brazilian legislation in Parobé and Sapucaia do Sul sites, located in the middle and in the lower courses of the Sinos River, respectively. The Caraá site, located in the upper river reach, presented all the physicochemical parameters in accordance with the allowed limits in both sampling periods. Comet assay in fish revealed genotoxicity in water samples collected in the middle course site in summer and in the three sites in winter when compared to control group. Thus, the physicochemical parameters indicated that the water quality of the upper course complies with the limits set by the national guidelines, and the ecotoxicological assessment, however, indicated the presence of genotoxic agents. The present study highlights the importance of combining water physicochemical analysis and bioassays to river monitoring.

## 1. Introduction

Rivers receive a huge amount of wastes from urban areas and industrial and agricultural activities [1]. Hence, water bodies will be contaminated with complex, ill-defined mixtures of chemicals and most freshwater organisms will be exposed, to varying degrees, to this contamination [2]. In this context, chemical analysis of water is not sufficient to assess their toxic potential for wildlife and humans. This is because the bioavailability, the biological activities, and interactions between different environmental chemicals are not completely understood and considered when hazard assessments and predictions of possible ecotoxicological effects are made based on concentrations alone [3]. Therefore, a simultaneous application of bioassays is a good complement to chemical analyses and a useful tool to establish the ecological effects to environment, as it provides the complete response of test organisms to all the compounds in the water [4]. 

Among the methods available to assess the many possible toxic effects caused by the chemicals present in the environment, the analysis of DNA alterations in aquatic organisms has been shown to be a highly suitable method for evaluating the genotoxic contamination of environments, being able to detect toxicity at low concentrations of contaminants in a wide range of species [5]. The pollution-induced genetic damage might cause adverse effects to species and affect the stability of ecosystems [6–8]. The comet assay or single cell gel electrophoresis is a rapid, sensitive, and relatively inexpensive method to study DNA damage in different cell types and has found wide application in genotoxicity evaluation in fish exposed to various xenobiotics in the aquatic environment [1, 9]. The comet assay is based on the ability of negatively charged fragments of DNA to be drawn through an agarose gel in response to an electric field. The extent of DNA migration depends directly on the DNA damage present in the cells [1]. 

The subtropical Sinos River is one of the main rivers located in Brazil's southernmost state, Rio Grande do Sul, and is an example of an impacted watercourse. The Sinos River basin presents an area of approximately 4,000 km^2^, which covers 32 municipalities, and provides drinking water for 1.2 million inhabitants and for one of the most important industrial centers in Brazil [10]. In the downriver direction, the river is divided into upper, middle, and lower courses, following a gradient of pollution. The Sinos River in the upper course has low demographic density and suffers moderate impacts from small amounts of agricultural waste and relatively little influence from domestic sewage. The middle course has a major impact with large areas used for rice cultivation and cattle ranching and has a larger number of inhabitants. In the lower course the demographic density and concentration of industries are high [10]. The most densely populated urban areas include three cities with more than 200,000 inhabitants (Novo Hamburgo, São Leopoldo, and Canoas), which are located in the lower course of the river. 

Studies by the State Environmental Protection Agency (Fundação Estadual de Proteção Ambiental, FEPAM) revealed high loads of domestic and industrial sewage in the lower course of the Sinos River [10]. Moreover, some chemical parameters in the middle course revealed that its water quality index was similar to that of the lower region of the basin [11]. Studies investigating the genotoxicity of water samples collected from the Sinos River have yielded both positive [12–14] and negative [14–17] results. 

In our previous investigation of genotoxicity of Sinos River, the results suggested that the sampling site near the river spring is not appropriate as reference site to fish genotoxicity bioassays [13]. Therefore, the aim of the present study was to evaluate the water quality of Sinos River through physicochemical parameters and genotoxic assay using the fish *Hyphessobrycon luetkenii *as organism test.

## 2. Materials and Methods

### 2.1. Sampling Sites

Water samples were collected in March and July 2008 (summer and winter, resp.) at three sampling sites: in the upper Sinos River course, near the river spring (town of Caraá: S 29°43′26′′ and W 50°16′46), in the middle course (town of Parobé: S 29°41′05′′ and W 50°50′52′′), and in the lower river course (town of Sapucaia do Sul: S 29°47′53′′ and W 51°11′24′′).

### 2.2. Fish Bioassay

Healthy juvenile specimens of indigenous fish species *Hyphessobrycon luetkenii* (Boulenger, 1887) (Characidae) were obtained from a local fish farm. Animals were randomly divided into four groups of about 12 specimens and placed in a 10 L aquarium, with well-oxygenated, dechlorinated tap water at 21°C ± 2°C, for a 96 h acclimatization period; they were then released into 10 L aquariums with undiluted water samples from each site within 4 h after sampling. Tap water was used as negative control group. The exposure period was 48 h with no food supply. 

### 2.3. Comet Assay

After the exposure period, the comet assay was performed on peripheral erythrocytes, according to Tice et al. [19]. Slides were precoated with normal melting point agarose. A mixture of 5 *µ*L of blood sample collected from caudal veins of fish with 95 *μ*L low melting point agarose (0.7%) was added to the slide and immediately covered with a coverslip and then kept for 10 min in a refrigerator to solidify. After solidification of the gel, coverslips were gently removed and the slides were immersed in cold, freshly made lysing solution (2.5 M NaCl, 100 mM EDTA, and 10 mM Tris, pH 10.2, to which 1% Triton X-100 and 10% DMSO had been added) and refrigerated at 4°C for 6–24 h. After the lysis, the slides were placed in a horizontal electrophoresis box side by side. The tank was filled with fresh electrophoresis buffer (300 mM NaOH and 1 mM EDTA, pH > 13) at 4°C. The liquid covered the slides, which were then left in the solution for 20 min before the power was turned on. Electrophoresis was performed at 25 V and 300 mA (~0.95 V/cm) for 20 min. The steps above were carried out under red light to avoid induction of DNA damage. After electrophoresis, the slides were gently removed from the tank, and neutralizing buffer (0.4 M Tris, pH 7.5) was added to the slides dropwise three times, letting it sit for 5 min each time. The slides were rinsed three times with distilled water, air-dried for at least 24 h, and then fixed and stained with silver stain according to Nadin et al. [20]. 

For evaluation of DNA damage, 100 cells per individual were analyzed under an optical microscope at 400x magnification. The analysis of the slides was conducted using the quality assurance criteria suggested by Tice et al. [19]. All slides were coded and examined blind by the same observer. The cells were visually scored according to tail length into five classes, from undamaged (class 0) to complete damage (class 4) [21]. Value was assigned to the different categories (from class 0 = 0 to class 4 = 4) and a damage index was calculated [(No. of class 0 cells × 0) + (No. of class 1 cells × 1) + (No. of class 2 cells × 2) + (No. of class 3 cells × 3) + (No. of class 4 cells × 4)] for each fish [22]. Therefore, the total score per individual ranged from 0 (all undamaged) to 400 (all maximally damaged). The damage frequency was calculated as the percentage of damaged cells (e.g., the sum of cells with damage classes 1 to 4).

### 2.4. Physicochemical Analysis

Immediately after collection, water samples were analyzed according to *Standard Methods for the Examination of Water and Wastewater* [23]. The following parameters were analyzed: pH, chemical oxygen demand (COD), five-day biological oxygen demand (BOD_5_), conductivity, chlorides, hardness, total nitrogen, ammoniacal nitrogen, phosphorus, aluminum, lead, chromium, copper, nickel, iron, zinc, sodium, total solids, dissolved solids, total volatile solids, nitrite, nitrate, turbidity, dissolved oxygen (DO), total coliforms, and fecal coliforms (*Escherichia coli*).

### 2.5. Statistical Analysis

Statistical analysis was performed using ANOVA, followed by Tukey multiple comparison test when appropriate. All analyses were carried out using the Statistical Package for the Social Sciences (SPSS) 15.0 for Windows, considering a significance level of *P* ≤ 0.05.

## 3. Results

The values of physicochemical variables measured in the three sampling sites during the summer and winter seasons are shown in Table 1. The values were compared to CONAMA Resolution 357/2005 by the Environment National Council [18] for classifying the national water bodies. In this legislation, the freshwater systems were allocated to five classes (from the class 1 to the class 4, water quality decreases and restrictions to water use as drinking supply increase) and upper limits were fixed for different water variables for each category (some of them for class 2 category are shown in Table 1). Freshwater systems within class 2 category may be used for human supply (after treatment), aquatic biological community protection, recreation, irrigation, aquaculture, and fishing activities.

Concerning the nineteen water parameters with reference values defined by CONAMA Resolution *n*. 357, higher values than standards were observed in sites Parobé and Sapucaia do Sul. Higher values were observed in Parobé for aluminum, iron, and fecal coliforms in the two sampling periods, for total phosphorus in summer, and for BOD in winter. The two water samples of Sapucaia do Sul showed BOD, conductivity, total phosphorus, iron, dissolved oxygen, and fecal coliforms values at odds with the CONAMA resolution, in addition to aluminum and copper in the winter sample. The Caraá site presented all the physicochemical parameters in accordance with the allowed limits in both sampling periods.

The results of damage frequency (%) and damage index estimated by the comet assay in the erythrocytes of *H. luetkenii* exposed to water samples from three sampling sites in the Sinos River and to tap water (control) are summarized in Table 2. In the summer period, damage frequency at Parobé site was significantly higher than that found at Caraá and in the control group; however, no differences in damage index were observed. The fish exposed to water samples collected at the three sampling sites in winter have shown significant increase in both damage index and damage frequency when compared to control group.

## 4. Discussion

Resolution *n*. 357 [18] is a guiding tool used to evaluate water quality in Brazil, and in the present study, values above those described in resolution were observed at sites Parobé and Sapucaia do Sul, located in the middle and lower courses of the Sinos River, respectively. Thus, water quality tended to decrease downstream as the river passes through the densely urbanized and industrial areas and receives discharge of treated and untreated domestic and industrial effluents [24]. According to the Sinos River Basin Management Committee, at present, domestic sewage is considered to be the major problem affecting water quality of the Sinos River because only 10% of domestic sewage is treated. The results obtained for dissolved oxygen indicated anoxic conditions of water in Sapucaia do Sul, the lower course site. Moreover, Parobé and Sapucaia do Sul showed fecal coliforms and BOD_5_ values higher than the limits, possibly as a consequence of high organic matter input from untreated domestic sewage discharge [25].

Apart from the domestic sewage, the Sinos River also receives pollutants from industrial and rural waste, which have been reported as a source of toxic and mutagenic effects on the environment [5, 26]. Studies on the genotoxicity of Sinos River basin have yielded conflicting results. Absence of genotoxic activity was found in the *Salmonella*/microsome test [15], the micronuclei analysis in the V79 cell line (Chinese hamster lung fibroblasts) [16], the wing somatic mutation and recombination test (SMART) in *Drosophila melanogaster* [17] and the *Allium cepa* test (micronucleus test) [14]. On the other side, Vargas et al. [15] have found genotoxic effects in the microscreen phage-induction assay and Lemos et al. [12] using *Salmonella*/microsome assay found indicative mutagenic response, including in the headwaters of a tributary of the Sinos River. Moreover, Nunes et al. [14] found positive results in V79 cell line (comet assay and micronucleus test).

Although the *in vitro* tests used in most studies assessing Sinos River genotoxicity are recommended methods to evaluate the mutagenic activity of environmental samples, such tests do not provide an assessment of genotoxicity in ecologically relevant organisms [27], such as fish species. In the first study in which Sinos River water genotoxicity was assessed using fish biomarkers, the comet assay has shown significant differences between sampling periods, with higher values in water samples collected at sites in the upper and middle course during the spring season [13]. The results suggested that sites near the river spring are not appropriate as reference sites to fish genotoxicity bioassays. In addition, based on the monitoring of physicochemical parameters of Sinos River water, Blume et al. [11] have concluded that the sampling sites in the upper course should no longer be used as a reference point in future studies.

In the present study, the water samples collected in the summer in the middle course site and in the winter in the three sites have shown genotoxicity in the peripheral blood of *H. luetkenii* when compared to negative control, confirming the results of other studies that have detected contamination of genotoxic compounds in the Sinos River basin, including the upstream course [12, 13]. Thus, the results of this study indicate that native fish species might be at risk for genotoxic damage in the Sinos River. However, the genotoxic potential of contaminants is an organism-specific and exposure-dependent phenomenon and interspecies variability for toxic responses as a result of differences in uptake, accumulation, metabolism, excretion, and DNA repair efficiency should be considered [8].

Many studies investigated the genotoxic effects of rural, industrial, and urban effluents; however, their relative contribution and comprehensive chemical characterization remain a major challenge [26, 28, 29]. Considering the low urban density and the economic activities in the upper Sinos River site (Caraá), this region suffers relatively little influence from domestic and industrial sewage and genotoxic pollutants might come from small farms and livestock. Contaminants in this region threaten the sustainability of one of the main basins of southern Brazil, responsible for water supply to approximately 1.6 million inhabitants, representing 17% of the population of the Rio Grande do Sul state [24]. Further studies in the upper Sinos River course should be conducted to identify sources of water contaminants so that measures can be taken to reduce or eliminate their negative effects.

## 5. Conclusion

Physicochemical parameters demonstrated decreasing water quality from upstream to downstream of the Sinos River and comet assay in fish showed that the river water may be contaminated with genotoxic pollutants in the upper, middle, and lower courses. Thus, despite good water quality of the upper river course, it should no longer be used as a reference site in future genotoxicity studies. Moreover, the results showed that complementing the physicochemical evaluation of river water with bioassays clearly permits acquisition of information that cannot be obtained from the measurement of water quality parameters.

## Figures and Tables

**Table 1:** Physicochemical parameters of water samples from different sites of Sinos River in the summer and winter of 2008.

Parameter	CONAMA	Caraá Upper course		Parobé Middle course		Sapucaia do Sul Lower course	
		Summer	Winter	Summer	Winter	Summer	Winter
BOD_5_, mgO_2_ L^−1^	5.0	<1.0	1	1	7*	7*	12*
COD, mgO_2_ L^−1^	—	8	2	10	7	15	34
Chlorides, mg L^−1^	250	3.1	2.9	2.9	3.5	12.2	10.7
Conductivity, *μ*S cm^−1^	100	32.75	29	69.05	68.45	142.85*	119.7*
Hardness, mgCaCO_3_ L^−1^	—	7	13	22	29	31	42
Total nitrogen, mg L^−1^	—	1.3	<0.5	2.28	<0.5	3.91	<0.5
Ammoniacal nitrogen, mg L^−1^	3.7	nd	<0.5	nd	<0.5	1.46	<0.5
Total phosphorus, mg L^−1^	0.1	nd	<0.012	0.105*	0.06	0.201*	1.075*
Aluminum, mg L^−1^	0.1	0.05	nd	0.59*	0.18*	0.05	2.0*
Lead, mg L^−1^	0.01	nd	nd	nd	0.0015	nd	0.004
Chromium, mg L^−1^	0.05	nd	nd	nd	nd	nd	0.04
Copper, mg L^−1^	0.009	nd	nd	0.002	nd	0.004	0.04*
Nickel, mg L^−1^	0.025	nd	nd	nd	nd	nd	0.01
Iron, mg L^−1^	0.3	0.08	0.23	1.93*	0.66*	1.13*	6.88*
Zinc, mg L^−1^	0.18	0.01	nd	0.01	nd	0.01	0.08
Sodium, mg L^−1^	—	5	5.4	6	6.9	13.4	14.1
Dissolved solids, mg L^−1^	500	10.5	77	35.5	91	17	110
Total solids, mg L^−1^	—	51.5	197	117.5	215	169	870
Total volatile solids, mg L^−1^	—	34.5	144	75.5	133	105	227
pH	6.0 to 9.0	7.46	7.06	7.21	7.28	6.89	6.89
Nitrates, mg L^−1^	10.0	1.09	0.12	2.83	1.42	2.18	1.28
Nitrite, mg L^−1^	1.0	nd	0.003	0.04	0.04	0.16	0.12
Turbidity, NTU	100	0.42	0.02	17.4	0.9	12.4	50.5
Dissolved oxygen, mg L^−1^	5.0	8.13	8.53	7.31	7.11	4.47*	2.03*
Total coliforms, NMP/100 mL	—	5012	697	101120	68930	56300	23100
Fecal coliforms, NMP/100 mL	1000	85	84	2690*	8620*	3000*	5200*

Values were obtained from one water sample. nd: not detected. *Values at odds with the CONAMA resolution *n*. 357/05 upper limits to class 2 water category [18].

**Table 2:** Damage frequency (the sum of cells with damage classes 1 to 4, in %) and damage index estimated by the comet assay (mean ± standard deviation) in the erythrocytes of *H. luetkenii* exposed to water samples from the Sinos River and to tap water (control).

Sampling site	Summer		Winter	
	Damage frequency (*n*)	Damage index	Damage frequency (*n*)	Damage index
Caraá (Upper course)	50.6 ± 9.3 (12)^a^	58.1 ± 14.5	66.3 ± 13.3 (11)^a^	69.5 ± 14.2^a^
Parobé (Middle course)	61.3 ± 6.3 (12)^b^	64.6 ± 7.4	62.9 ± 16.0 (8)^a^	65.3 ± 17.9^a^
Sapucaia do Sul (Lower course)	53.3 ± 10.4 (12)^ab^	60.8 ± 16.0	59.6 ± 11.5 (11)^a^	62.5 ± 15.0^a^
Control	47.1 ± 11.5 (12)^a^	51.1 ± 14.2	40.4 ± 16.0 (8)^b^	40.4 ± 16.0^b^

*P *	0.005	0.107	0.002	0.002

The number of fish analyzed in each sample is presented between brackets. *P* values referring to ANOVA between sampling sites and respective control group (^a,b^values with different letters have significant difference in the same column).
